# Use of 3D-Printed Models and Augmented Reality in Medical Student Education of Congenital Heart Disease: Randomized Controlled Trial

**DOI:** 10.2196/85967

**Published:** 2026-05-05

**Authors:** Tyler Langenfeld, Gabriel N Bahrami, Yea-Lyn Pak, Susanne Wish-Baratz, Yuxi Zhu, Arpit Agarwal

**Affiliations:** 1 Department of Pediatrics, Rainbow Babies & Children's Hospital, Cleveland, OH, United States; 2 Department of Pediatric Cardiology, Rainbow Babies & Children's Hospital, 2101 Adelbert rd, Cleveland, OH, 44106, United States, 1 347-277-3021; 3 School of Medicine, Case Western Reserve University, Cleveland, OH, United States

**Keywords:** medical education, augmented reality, 3D models, congenital heart disease, teaching modalities

## Abstract

**Background:**

Three-dimensional modalities are increasingly being used as adjuncts for medical trainees learning about complex anatomical concepts, such as congenital heart disease.

**Objective:**

This study aimed to evaluate the use of 2 such modalities, 3D-printed models, and augmented reality (AR), in improving medical students’ understanding and knowledge retention of congenital heart disease when compared to traditional teaching methods.

**Methods:**

A prospective cohort pilot study was performed with 26 first-year medical students. Students were randomly assigned to receive a 30-minute teaching session using traditional slide-based lecture, 3D-printed model, or AR. Participants completed a 16-question pretest consisting of 4 basic general cardiology questions and 6 questions each regarding the anatomy and physiology of tetralogy of Fallot and hypoplastic left heart syndrome. Participants completed a posttest immediately following the teaching session, as well as a delayed posttest 3 weeks later.

**Results:**

When comparing overall and subsection posttest scores, the AR group obtained perfect immediate posttest scores at a significantly increased rate compared to the lecture and 3D model groups (6/9, 67% vs 1/8, 13% and 1/9, 11%, respectively; large effect size Cramér V=0.57; *P*=.02). Participants in the lecture group reported difficulty understanding cardiac anatomy and physiology using only 2D diagrams, whereas those in the 3D-printed model and AR groups almost unanimously reported improved visualization of complex cardiac defects, which enhanced their understanding.

**Conclusions:**

Due to the visuospatial benefits of 3D-printed models and AR, there is potential for use in medical education to improve students’ knowledge of complex anatomical and physiological concepts. Students who received teaching using 3D-printed models or AR overwhelmingly reported improved 3D visualization of congenital cardiac defects compared to those who were taught via lecture. Additionally, AR and 3D-printed models offer practical opportunities for implementation into medical education curricula as both adjunct and stand-alone teaching modalities.

## Introduction

Determining the best methods of content delivery has become a hallmark of education research in recent years. Aligning teaching methods with students’ preferred “learning styles,” such as through visual, aural, read or write, and kinesthetic assessment, is popular among educators, though it has not been shown to improve learning [1,2]. The use of “flipped classrooms” is also growing in popularity, with some estimates showing over half of US colleges use this model [3]. However, it has failed to show empirical evidence that it improves learning [4]. The primary aim across these approaches is to foster active learning [4].

Medical education is no stranger to these alternative learning models, but it carries the added complexity of encompassing concepts that are both highly visual (eg, anatomy) and rather abstract (eg, immunologic processes). Therefore, medical school education routinely implements a variety of methods and modalities to best deliver a given topic [5]. Gross anatomy is traditionally taught with cadaveric dissection and 2D visual representations, while topics such as physiology and biochemistry rely more heavily on textbook readings and lectures. Strong visuospatial skills are required to understand complex human anatomy. Students who face difficulties learning anatomy often do so because they struggle to visualize the structures in 3D [6]. Therefore, students who develop spatial abilities are better able to learn anatomy [6]. With the goal of enhancing students’ visuospatial skills in mind, various modalities and methods have been explored to augment traditional slide-based lectures. Two modalities that can provide 3D context to anatomy lectures and improve visual conceptualization are 3D-printed models and augmented reality (AR).

Slide-based lectures have been the standard for teaching modalities in higher education for many years. Its popularity is in part due to its ability to condense and organize large amounts of information as well as add visuals such as graphs, charts, and animations to supplement the lecture. Creating a slide-based presentation offers a relatively small learning curve, making it accessible to a wide majority of instructors. Furthermore, the slides are easily stored and disseminated to students, allowing for review of the material at any time. However, this method has several drawbacks. This style of lecture often promotes passive learning and leads to less engaged learners [7]. Slide presentations are also limited to 2D visuals and are thus not ideal for topics that require understanding of complex 3D structures.

3D-printed models are one of the potential solutions aimed at bridging this learning gap. With recent technological improvements, it is now possible to produce life-sized, flexible 3D replicas of real human organs. This provides learners with hands-on experience with complex structures and enables them to manipulate the models in a way that is not possible with modalities other than cadavers. Despite the technological advancements, the cost of producing 3D models is still high. It can cost as much as US $65,000 for a printer, US $15,000 for the software, and another US $400 per hour to obtain the requisite computed tomography scans from which the models will be based [8]. Accurate use of the printers requires trained personnel, which further limits their use. Despite their portability, the excessive cost of the models limits student access outside the classroom, reducing opportunities for review.

AR is among the latest technologies being explored in medical education. This modality allows learners to visualize complex anatomy in 3D but does not offer the tangible element of 3D-printed models. AR offers an immersive and interactive experience that allows users to interact with the projected images without the physical constraints imposed by a 3D model. With the appropriate software, learners can “take apart,” zoom-in, and overlay models as they see fit. Being able to effectively use AR requires a comfortable level with its operation for all involved, which leads to a relatively steep learning curve when compared to other modalities. It requires personnel able to create the digital files of the teaching material and instructors who are familiar with the technology. Students unfamiliar with AR due to its novelty may experience a learning curve with the controls before using it as a learning method. Finally, AR can induce several side effects, such as headaches and motion sickness, which can adversely affect the learning experience [9].

Each learning modality has clear benefits and limitations, and with increasing availability, each has the potential to play a role in the future of medical education in some capacity [10-17]. This study aims to evaluate and compare the impact of traditional slide-based lecture, 3D-printed models, and AR on undergraduate medical students’ understanding of complex anatomical and physiologic concepts as well as their subjective experiences with the modalities.

## Methods

### Overview

A prospective randomized controlled pilot study was conducted with first-year medical students at Case Western Reserve University in Cleveland, Ohio. A standardized curriculum was developed to ensure equivalent subject matter was covered in each group. A slide-based lecture was created using Microsoft PowerPoint and included text, static 2D figures, and basic 2D animations but did not include any videos or other media. A 16-question test was created consisting of 4 basic general cardiology questions and 6 questions each regarding the anatomy and physiology of tetralogy of Fallot (TOF) and hypoplastic left heart syndrome (HLHS). The test questions were reviewed by 3 pediatric cardiologists for technical accuracy. The questions were evaluated for face validity by 5 peers and pilot-tested on 10 participants, comprising medical residents and upper-level medical students. Other psychometric properties, including reliability and standardized administration, were reviewed with an expert in medical education and survey-based research.

**Figure 1.**
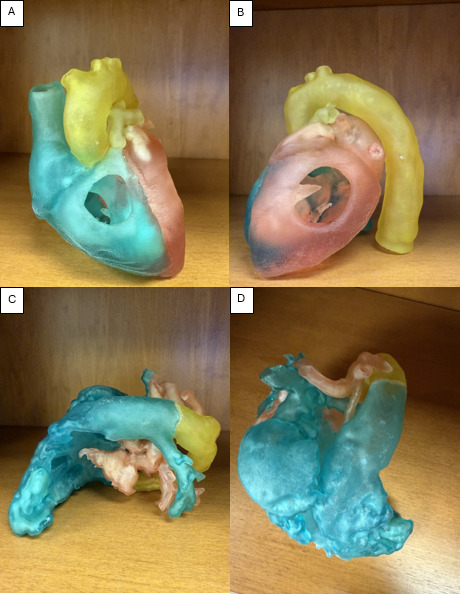
Three-dimensional–printed heart models: (A) right-sided view of tetralogy of Fallot (TOF), (B) left-sided view of TOF, (C) left-sided view of hypoplastic left heart syndrome (HLHS), and (D) overhead view of HLHS.

**Figure 2.**
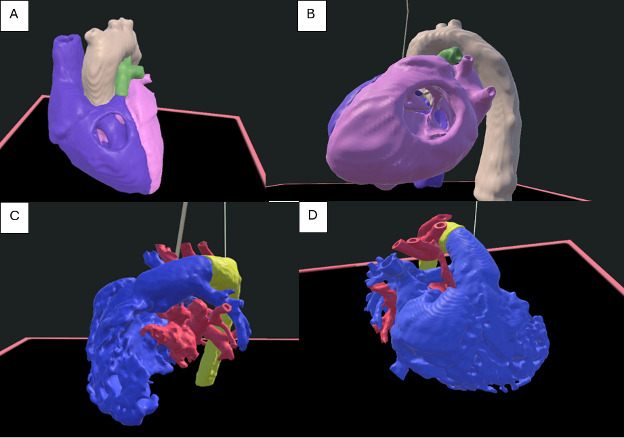
Augmented reality heart models: (A) right-sided view of tetralogy of Fallot (TOF), (B) left-sided view of TOF, (C) left-sided view of hypoplastic left heart syndrome (HLHS), and (D) right-sided view of HLHS.

Using the Unity development platform, a new AR application was developed to allow students an immersive experience using the HoloLens (Microsoft) headset. Images were sourced from cardiac computed tomography angiography images of deidentified patients with normal heart anatomy, TOF, and HLHS. The computed tomography angiography images were segmented using Elucis virtual reality software (Realize Medical) to create high-fidelity 3D anatomic models. The models were printed in 3 colors to better represent and differentiate the cardiac anatomy: blue to represent right-sided heart structures, red for left-sided structures, and yellow for pathologic structures such as a patent ductus arteriosus (Figure 1). The virtual 3D anatomic models were then converted to compatible file formats and uploaded to the Unity app and subsequently downloaded on Microsoft HoloLens to deliver the AR teaching (Figure 2). The same files were used to create a 3D-printed model using a Stratasys Medical 3D printer.

Participants were randomly assigned to one of three groups: (1) traditional slide-based teaching, (2) hands-on instruction with 3D-printed heart models, and (3) AR instruction using HoloLens headsets. Simple randomization was performed, with participants assigned a number from 1 to 3 corresponding to their respective study groups. Numbers were given sequentially in order of participant arrival to the intervention to ensure similar size among the groups. The study was nonblinded as study team members provided the teaching sessions, and participants knew which modality they were receiving. However, the study team member who graded the questionnaires was blind to which modality was received.

Participants completed the 16-question pretest prior to taking part in a 30-minute teaching session using their assigned modality. Sessions were completed simultaneously, and each group used only the modality to which they were assigned (ie, the 3D model and AR groups did not provide corresponding teaching slides). Each group received teaching on the same topics, which included basic anatomy and physiology of the normal heart, TOF, and HLHS. The study team members who conducted the 3D model and AR teaching tailored their sessions based on the prepared slides to ensure similar content was being taught across all 3 groups. Each session was reviewed by a group of 3 cardiologists during a practice session to ensure each group received an equal amount of information.

Upon completion of the teaching session, participants completed a posttest questionnaire consisting of the same 16 questions. A delayed posttest was conducted 3 weeks later with the same questionnaire to evaluate the participants’ knowledge retention. The delayed posttest also contained qualitative questions regarding their experience with the teaching session and use of the assigned modality. Confidentiality was ensured by not collecting any personally identifiable information from participants. Pretests and posttests were linked using participant-created codes that did not contain personal identifiers.

The test was divided into 3 sections: general cardiology, TOF, and HLHS. For each participant, 4 scores (general score, TOF score, HLHS score, and total score) were recorded at the pretest, posttest, and delayed posttest. Improvement scores were calculated by subtracting the pretest score from the posttest and delayed posttest scores. A perfect score was defined as achieving the full total score (16 points) on the posttest and was recorded for each modality.

Descriptive statistics were used to summarize the distribution of participants’ scores. Medians with IQRs were reported for continuous variables, and frequencies with proportions were reported for categorical variables. To compare scores across modalities, the Kruskal-Wallis rank-sum test and Fisher exact test were applied to continuous and categorical variables, respectively.

To enhance the power to detect differences in score improvements from the pretest to the posttest and delayed posttest across the 3 modalities, a difference-in-differences (DID) analysis was performed. This approach assessed the impact of each modality by comparing score changes over time. The DID model included an interaction term between modality and time point. To account for participant-level clustering, a random intercept was included using a mixed-effects model. All statistical analyses were conducted using R (version 4.3.3; R Foundation for Statistical Computing).

### Ethical Considerations

Institutional review board approval was obtained from Case Western Reserve University as STUDY20240627. Students were recruited to participate in the study through emails and in-person announcements. Participants were provided with written and verbal explanations of the study procedures. Informed consent was obtained prior to the initial teaching session, and participants were informed of their ability to opt out of the study at any point. Participation was voluntary, and participants did not receive financial compensation. Confidentiality was ensured by not collecting any personally identifiable information from participants. Pretests and posttest were linked using participant-created codes that did not contain personal identifiers.

## Results

**Figure 3.**
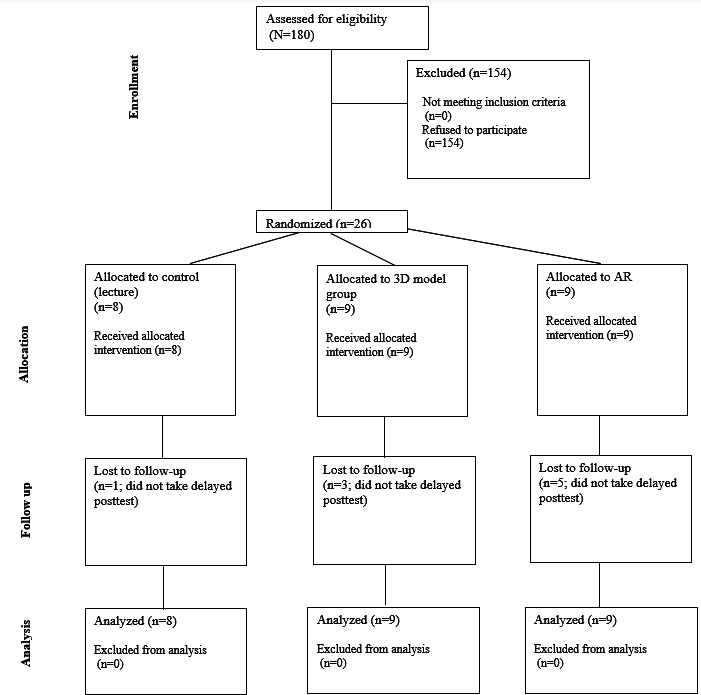
CONSORT (Consolidated Standards of Reporting Trials) diagram. AR: augmented reality.

**Figure 4.**
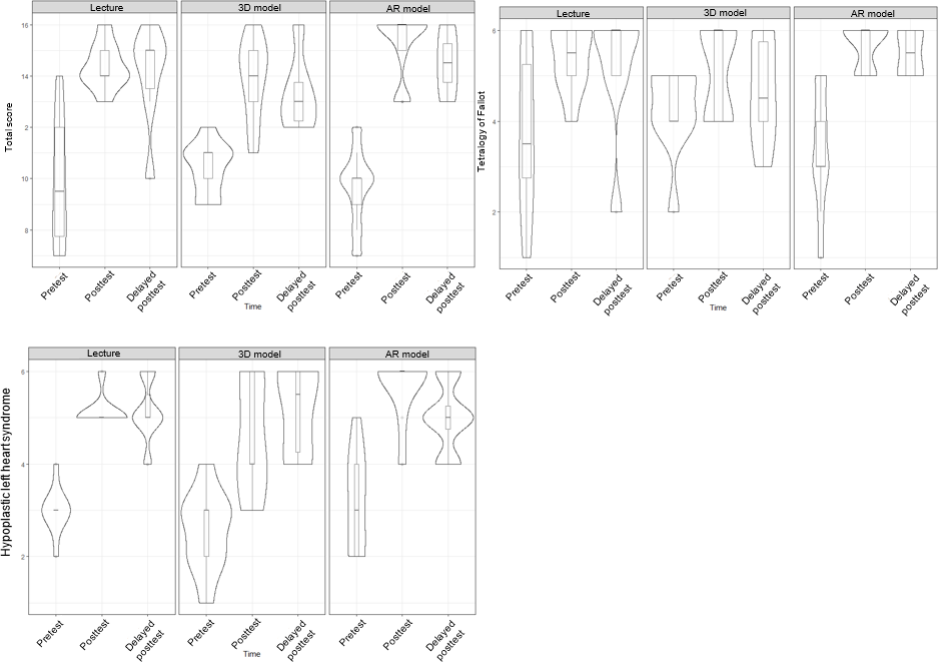
in Figure Score distribution at 3 test time points for each modality. AR: augmented reality.

**Figure 5.**
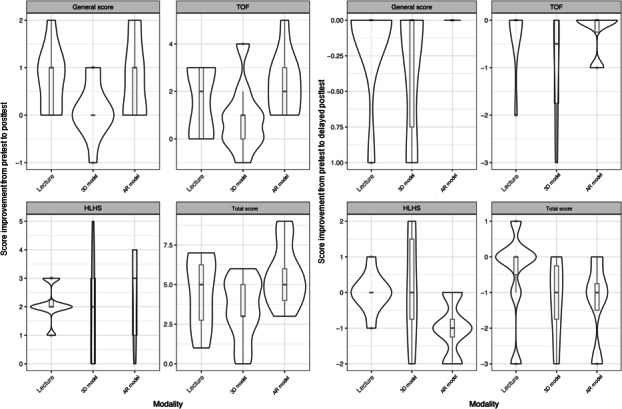
Difference in immediate and delayed posttest scores from pretest for each modality. AR: augmented reality; HLHS: hypoplastic left heart syndrome; TOF: tetralogy of Fallot.

A total of 26 first-year medical students participated in the study. In total, 8 (31%) participants were instructed by slide-based lecture, 9 (35%) were taught using 3D printed models, and 9 (35%) used the HoloLens AR goggles (Figure 3). Of the 26 participants, 17 (65%) completed the delayed posttest. Table 1 shows the median total and individual category scores, as well as the IQRs for each modality. Figure 4 shows violin plots of these scores. Figure 5 shows the improvement in posttest and delayed posttest scores compared to pretest scores for each modality.

**Table 1.** Median (IQR) scores by modality.

Questions		Lecture, median (IQR)	3D model, median (IQR)	AR^a^, median (IQR)	*P* value
Pretest					
General		3 (2.75 to 4)	4 (3 to 4)	3 (3 to 4)	.20
TOF^b^		3.5 (2.75 to 5.25)	4 (4 to 5)	3 (3 to 4)	.40
HLHS^c^		3 (3 to 3)	3 (2 to 3)	3 (2 to 4)	.40
Total		9.5 (7.75 to 12)	11 (10 to 11)	10 (9 to 10)	.60
Posttest					
General		4 (4 to 4)	4 (4 to 4)	4 (4 to 4)	.80
TOF		5.5 (5 to 6)	6 (4 to 6)	6 (5 to 6)	.50
HLHS		5 (5 to 5)	4 (4 to 6)	6 (6 to 6)	.05
Total		14 (14 to 15)	14 (13 to 15)	16 (15 to 16)	.10
Improvement from pretest					
General		1 (0 to 1)	0 (0 to 0)	1 (0 to 1)	.13
TOF		2 (0 to 3)	1 (0 to 1)	2 (1 to 3)	.12
HLHS		2 (2 to 2.25)	2 (0 to 3)	3 (3 to 4)	.80
Total		5 (2.75 to 6.25)	3 (3 to 5)	5 (4 to 6)	.20
Delayed posttest					
General		4 (3.5 to 4)	3.5 (3 to 4)	4 (4 to 4)	.30
TOF		6 (5 to 6)	4.5 (4 to 5.75)	5.5 (5 to 6)	.50
HLHS		5 (5 to 5)	5.5 (4.25 to 6)	5 (4.75 to 5.25)	>.99
Total		15 (13.5 to 15)	13 (12.25 to 13.75)	14.5 (13.75 to 15.25)	.40
Delayed posttest improvement					
General		1 (0 to 1)	0 (−0.75 to 0.75)	0.5 (0 to 1.25)	.30
TOF		1 (0.5 to 3)	0.5 (0 to 1)	1 (0.75 to 1.50)	.30
HLHS		2 (1.5 to 3)	3 (2.25 to 3)	3 (2.5 to 3)	.40
Total		5 (3 to 6)	3 (3 to 3.75)	4.5 (3.25 to 5.75)	.50

a AR: augmented reality.

b TOF: tetralogy of Fallot.

c HLHS: hypoplastic left heart syndrome.

**Figure 6.**
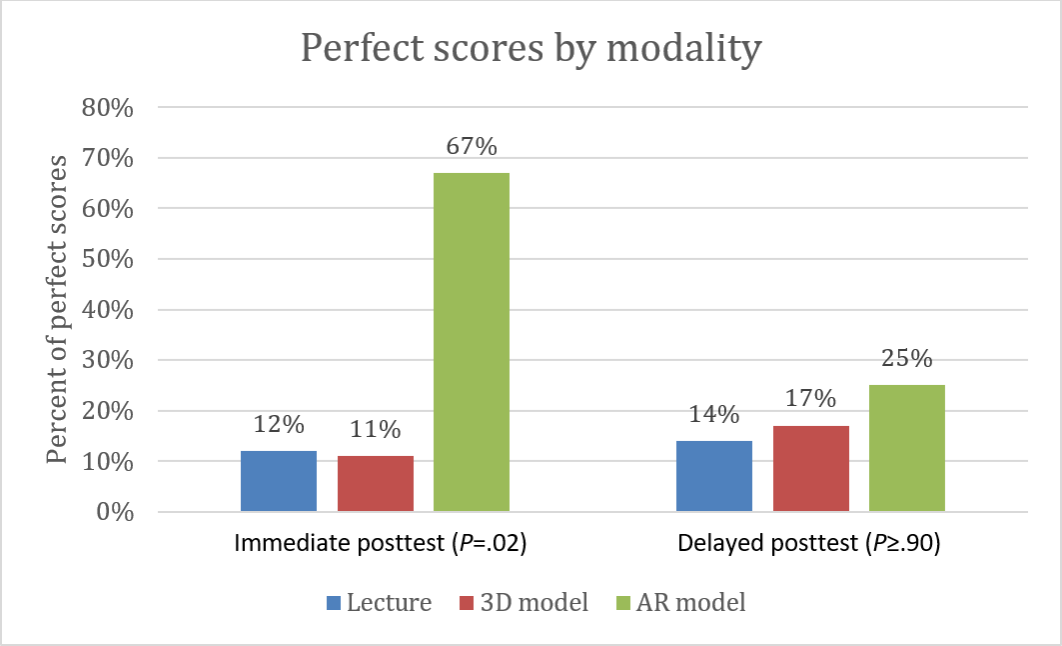
Perfect scores obtained on posttests by modality. AR: augmented reality.

There was no statistically significant difference in total median scores of pretest and posttest between the 3 modalities. There were also no statistically significant differences in subsection scores between modalities. The AR group (median 6, IQR 6‐6) demonstrated higher immediate posttest scores on the HLHS questions compared with both the lecture model (median 5, IQR 5‐5) and the 3D model (median 4, IQR 4‐6; Kruskal-Wallis *χ^2^*_2_= 5.9; *P*=.05). Participants exposed to AR teaching obtained a perfect immediate posttest score at a significantly increased rate (6/9, 67%) compared to the lecture (1/8, 13%) and 3D model (1/9, 11%) groups (large effect size Cramér V=0.57; *P*=.02), though this benefit was not seen in the delayed posttest (Figure 6).

The results of the DID analysis are shown in Tables2  3. A statistically significant difference in total score improvement from pretest to posttest was observed between the AR and 3D models (mean difference 2.11, 95% CI 0.34-3.88; *P*=.02), indicating that the AR model led to a greater improvement in total scores compared to the 3D model. For the general category scores, participants in the lecture group showed a 0.64-point greater improvement than those in the 3D group (95% CI −0.02 to 1.30; *P*=.06). Additionally, participants in the AR group demonstrated a statistically significant 0.67-point greater improvement compared to the 3D group (95% CI 0.03-1.31; *P*=.05).

**Table 2.** Comparison of score improvements from pretest to posttest and delayed posttest across 3 modalities, using the 3D model as the reference.

	Lecture model		AR^a^ model	
	Mean difference (95% CI)	*P* value	Mean difference (95% CI)	*P* value
Improvement in scores from posttest to pretest				
General	0.64 (−0.02 to 1.3)	.06	0.67 (0.03 to 1.31)	.05
TOF^b^	0.74 (−0.45 to 1.92)	.23	1.44 (0.29 to 2.60)	.02
HLHS^c^	0.12 (−1.04 to 1.29)	.83	0.44 (−0.68 to 1.57)	.44
Total score	1.06 (−0.77 to 2.88)	.26	2.11 (0.34 to 3.88)	.02
Improvement in scores from delayed posttest to pretest				
General	0.76 (0.05 to 1.48)	.04	0.99 (0.21 to 1.78)	.02
TOF	1.13 (−0.17 to 2.43)	.10	1.58 (0.13 to 3.02)	.04
HLHS	−0.47 (−1.72 to 0.78)	.46	−0.83 (−2.18 to 0.52)	.23
Total score	1.33 (−0.66 to 3.32)	.20	1.79 (−0.40 to 3.99)	.12

a AR: augmented reality.

b TOF: tetralogy of Fallot.

c HLHS: hypoplastic left heart syndrome.

**Table 3.** Comparison of score improvements from pretest to posttest and delayed posttest across 3 modalities, using the lecture model as the reference.

	3D model		AR^a^ model	
	Mean difference (95% CI)	*P* value	Mean difference (95% CI)	*P* value
Posttest to pretest				
General	−0.64 (−1.3 to 0.02)	.06	0.03 (−0.63 to 0.69)	.93
TOF^b^	−0.74 (−1.92 to 0.45)	.23	0.71 (−0.48 to 1.9)	.25
HLHS^c^	−0.13 (−1.29 to 1.04)	.83	0.32 (−0.84 to 1.48)	.59
Total score	−1.06 (−2.88 to 0.77)	.26	1.06 (−0.77 to 2.88)	.26
Delayed posttest to pretest				
General	−0.76 (−1.48 to −0.05)	.04	0.23 (−0.54 to 1.01)	.56
TOF	−1.13 (−2.43 to 0.17)	.10	0.44 (−0.98 to 1.86)	.54
HLHS	0.47 (−0.78 to 1.72)	.46	−0.37 (−1.7 to 0.97)	.60
Total score	−1.33 (−3.32 to 0.66)	.20	0.47 (−1.7 to 2.63)	.68

a AR: augmented reality.

b TOF: tetralogy of Fallot.

c HLHS: hypoplastic left heart syndrome.

**Figure 7.**
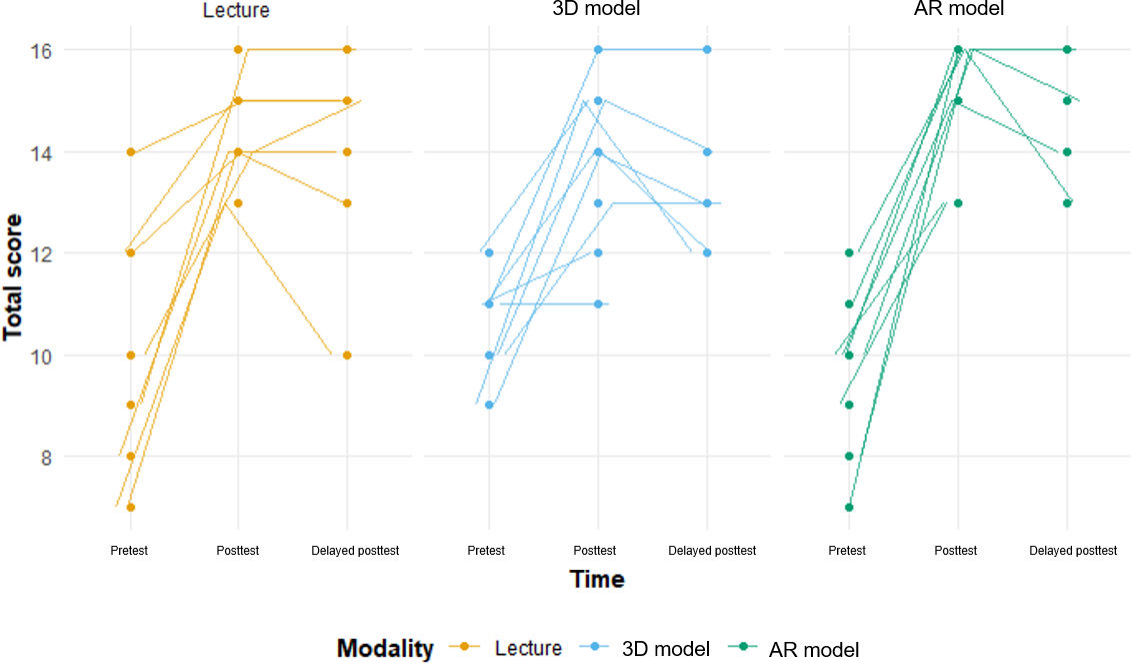
Individual trajectories of test scores across pretest, posttest, and delayed posttest. AR: augmented reality.

**Figure 8.**
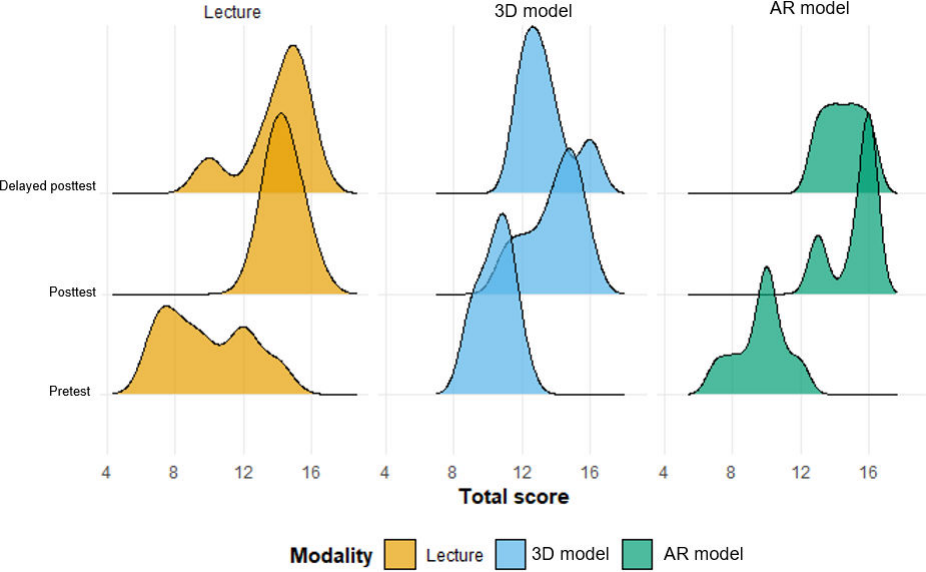
Temporal changes in score distributions across educational modalities. AR: augmented reality.

As shown in Figure 7 (individual) and Figure 8 (overall), the AR model demonstrated greater score improvement at the posttest compared to both the lecture group (mean difference 1.06, 95% CI –0.77 to 2.88; *P*=.26) and the 3D model group (mean difference 2.11, 95% CI 0.34-3.88; *P*=.02).

However, both the AR model (mean difference –0.59, 95% CI –2.75 to 1.57; *P*=.60) and the 3D-printed model (mean change –0.27, 95% CI –2.26 to 1.72; *P*=.79) showed a greater decline after the posttest when compared to the lecture group.

Participants’ subjective experiences were categorized by modality. Those who participated in the slide-based lecture reported the presence of diagrams and familiarity with the method as being most helpful in understanding the material. Participants in both the 3D-printed model and AR groups reported that improved visualization of cardiac defects benefited their learning, with the ability to physically handle structures providing an additional advantage for the 3D-printed models. A common drawback reported by lecture participants was a lack of 3D or hands-on experience. Multiple participants expressed difficulty grasping the anatomy and physiology using only 2D diagrams. Those in the 3D-printed model group noted difficulty manipulating the model to visualize internal structures. Some also reported a learning curve due to little or no prior experience with physical models. The primary downside noted by the AR group was intermittent glitches in the software.

## Discussion

### Principal Findings

In this pilot study of first-year medical students, we compared traditional slide-based lectures, 3D-printed models, and AR for teaching congenital heart disease. While we found no statistically significant data to suggest superiority of any one modality over another in relation to overall test scores, several important trends emerged. Students in the AR group achieved perfect immediate posttest scores at a significantly higher rate (*P*=.02) than those in the lecture or 3D model groups and demonstrated greater score improvements from pretest to posttest, particularly in total scores and TOF questions. DID analysis confirmed a significant advantage of AR over 3D models in immediate knowledge gain, though this did not persist with the delayed posttest. This finding may be partially explained by the small sample size, as discussed later, but it may also suggest that a single session using 3D models and AR is insufficient to produce significant improvements in retention. Subjectively, the 3D-printed model and AR groups reported that 3D visualization improved their understanding, while lecture participants reported greater difficulty conceptualizing structures using 2D diagrams.

Slide-based lectures remain a foundational component of medical education due to their efficiency and accessibility. However, their 2D format falls short when it comes to concepts that require a high degree of visuospatial understanding. This has led to the introduction of modalities such as 3D-printed models and AR into medical education curricula. In our study, students reported improved visualization, supporting the notion that 3D modalities may help address the gaps left by traditional teaching methods. Previous studies have compared 3D-printed models or AR to traditional methods [10,12,13,17]. Studies prospectively comparing all 3 modalities are, however, very limited.

Although the 3D-printed model and lecture groups showed no significant differences in total scores or score improvement, the students exposed to AR demonstrated statistically significant score improvement in multiple areas. Participants using the 3D-printed models and AR almost unanimously reported improved visualization of complex cardiac defects as a key benefit of the modality, whereas those in the lecture group felt that the lack of a 3D component hindered their understanding. This suggests that students recognize the importance of 3D modalities in conceptualizing complex anatomy and physiology.

Participants in this study experienced teaching exclusively on their assigned modality, that is, those who were taught using 3D-printed models or AR did not receive any supplementary slide-based presentation. White et al [10] performed a similar study comparing 3D-printed models to lectures in pediatric residents’ understanding of ventricular septal defect and TOF, demonstrating improved understanding of TOF in the 3D-printed model group. However, in their study, both groups were given the same lecture, with the 3D-printed model incorporated into the intervention group’s lecture. Importantly, even without accompanying slide-based lectures, the 3D-printed model and AR groups in this study performed comparably, or even superiorly, to the traditional teaching methods. These modalities are unlikely to fully replace traditional teaching methods; however, these findings suggest they can serve as a powerful adjunct. 3D modalities can fill visuospatial gaps left by traditional teaching, leading to more complete learning of complex anatomical and physiologic concepts such as congenital heart disease. Further investigation is therefore needed to evaluate the efficacy of incorporating these 3D modalities into traditional teaching methods.

AR and 3D-printed models can be feasibly incorporated into medical school curricula in several ways, as each has features that make it suitable for both classroom and laboratory settings. In the classroom setting, 3D-printed models can serve as a reference during traditional lectures. Students would be able to augment what they are learning from words, charts, and diagrams with a greater visuospatial component. These models can also serve as an adjunct in the cadaver laboratory as they can be compared to normal and diseased specimens as well as show anatomic variation within specific pathologies. Use of 3D-printed models allows for exposure to a wider variety of pathologies and structural anomalies than what can be feasibly achieved with cadavers. This adjunctive benefit is supported by a study by Aytaç et al [18], which demonstrated improved cardiac anatomy test scores in students taught using “plastinated” heart models (a process of dehydrating and fixing specimens) in addition to traditional formalin-fixed specimens, compared with those who received only formalin-fixed specimens.

Exposure to cardiac pathology specimens is usually limited by available cadavers or access to a specimen registry. 3D-printed models greatly increase the accessibility of physical representations of cardiac pathologies to students and allow for more hands-on learning. Furthermore, where cadaveric dissection is limited to sessions within the laboratory, 3D-printed models have no location restriction, which allows students to spend more time with the content material. Despite high initial costs, 3D-printed models can ultimately be more cost-effective than cadavers due to their durability and ability to be reused multiple times [8]. AR offers similar benefits to 3D-printed models, including improved access to visualization of cardiac pathologies and the ability to be used outside traditional laboratory or classroom settings. While 3D-printed models can be used simultaneously with traditional lecture, using AR simultaneously may be visually overstimulating and make it difficult to gain the desired effect. However, AR has several advantages over 3D-printed models. The first is the greater amount of information that can be conveyed in the AR space. AR cannot only display cardiac anatomy but also convey dynamic information such as blood flow, oxygen saturation, and pressures, which are essential for understanding key physiological concepts. The second is the lack of physical constraints inherent in 3D-printed models. Students are able to view cardiac anatomy in multiple ways, including interior structures that can be difficult to assess with physical models. They are also better able to view small structures, due to the ability to zoom in, with AR than in 3D-printed models or cadaveric specimens. Because of these qualities, using AR in stand-alone sessions can be a practical supplementation following traditional lectures.

The benefits of supplemental, interactive learning approaches have previously been explored. A recent study by Ogut et al [19] provided medical students with a supplemental course on cross-sectional anatomy of various organ systems with clinical case correlates using computed tomography and assessed their subjective experience. Students reported an overall positive experience with the course and believed it improved their learning, primarily with regard to integrating anatomical knowledge with clinical practice. Similar to the aforementioned study, our findings suggest that students favor interactive, visual methods for learning anatomical concepts.

### Limitations

There are several limitations that affect this study’s reliability and generalizability. The questionnaire used as the study’s assessment tool has only been pilot-tested and has therefore not been validated through peer review. The statistical power of the study was limited by a small sample size (n=26) and a high attrition rate (9/26, 34.6%). This is evident in the lack of significance in the posttest scores, where previous improvements in the 3D model and AR group appeared to diminish. The follow-up time of this study was 3 weeks, which limits conclusions about retention over longer periods that would be useful in medical education, such as board exam preparation.

The study population may present generalizability limitations as well. Participating medical students were from a single class at a single medical school. Students in this study were accustomed to AR technology as it is used routinely in their core curriculum. Therefore, they were not inhibited by the steep learning curve often associated with these devices. Conversely, they had little to no experience with 3D-printed models as a learning method. This introduces the possibility of familiarity bias affecting both objective and subjective outcomes. It is not surprising that students may gain relatively less understanding of a new and complex topic when it is presented in an unfamiliar format. Students appeared highly engaged with this modality, as many chose to spend additional time interacting with the models after completing the study. Therefore, the relatively smaller improvement following teaching with 3D models may be more a result of comfortability with the modality than the efficacy of the modality itself. Further studies involving participants who use 3D models as part of their standard curriculum and those unfamiliar with AR are warranted. Finally, this study focused on a narrow content topic of anatomy and physiology of 2 cardiac disease processes. This, however, does not limit the generalizability of this study’s findings, as cross-sectional imaging of any structure can be converted into 3D models and used for teaching purposes. AR, for instance, has been studied in numerous anatomical topics from skull to kidneys [16,17,20]. Therefore, advanced 3D visualization modalities can be applied to any body part or structural disease process and used for teaching complex anatomy to medical students.

### Conclusions

With advances in technology and the increased accessibility of these resources, we are seeing a paradigm shift toward the increasing use of 3D modalities in medical education and training. Students who received teaching using 3D-printed models or AR overwhelmingly reported improved 3D visualization of congenital cardiac defects compared to those who were taught via lecture. Owing to their visuospatial benefits, these modalities may serve as effective tools in medical education to improve student comprehension of complex anatomical and physiologic concepts. This study provides insight into the feasibility, benefits, and limitations of 3D modalities in medical education and emphasizes the importance of larger-scale studies to guide their integration into medical education and maximize educational impact.

## Supplementary material

10.2196/85967 Checklist 1 CONSORT-eHEALTH checklist (V 1.6.1).

## Abbreviations

AR: augmented reality
DID: difference-in-differences
HLHS: hypoplastic left heart syndrome
TOF: tetralogy of Fallot
